# Neurovascular Complications Associated With Clavicle Fractures: A Report of Three Cases and Recommendations

**DOI:** 10.7759/cureus.74506

**Published:** 2024-11-26

**Authors:** Ahmad Almigdad, Amar Malhas

**Affiliations:** 1 Department of Orthopedics, Royal Medical Services, Amman, JOR; 2 Department of Orthopedics, Royal Berkshire NHS Foundation Trust, Reading, GBR

**Keywords:** brachial plexus, clavicle, fracture, subclavian vessels, thoracic outlet syndrome

## Abstract

Neurovascular complications associated with clavicular shaft fractures can manifest at presentation, develop gradually over time, or potentially be iatrogenically induced. Conducting a thorough neurovascular examination and, when warranted, pursuing further investigation through modalities such as CT angiogram, MRI, and nerve conduction studies (NCS) are crucial for early diagnosis and pre-operative planning. This comprehensive approach enhances patient outcomes by facilitating timely intervention and addressing any underlying neurovascular issues associated with the fracture. Delayed fixation raises the risk of brachial plexus injury, highlighting the importance of timely intervention. This study aims to explore neurovascular complications associated with clavicle fractures through case presentations and provide management recommendations.

## Introduction

Clavicle fractures, accounting for up to 5% of adult fractures, have historically been treated non-operatively due to satisfactory outcomes with conservative methods. However, recent findings suggest superior results with surgical fixation, prompting greater interest in surgical fixation for selected patient populations [1]. The key to clavicle fracture management is identifying those who will have a poor outcome due to malunion or nonunion. Contributing factors include high-energy injury, comminution, shortening more than 2 cm, greater than 100% displacement, smokers, and females [2]. The connection between clavicle fractures and brachial plexus injury is widely recognized [3,4], often seen after high-energy traction injuries in the supraclavicular region [5,6]. Here, the clavicle fracture is more of an accompanying factor rather than a direct cause. Direct trauma to the brachial plexus from fragments of the clavicle is much less common and typically affects the infraclavicular portion of the plexus in only 1% of cases [7].

High-level studies investigating neurovascular injuries associated with clavicle fractures are limited, primarily due to the infrequency of such injuries. Nevertheless, several case reports and small series highlight the potential seriousness of these complications. Direct damage to major vessels and the brachial plexus may occur either at the time of trauma or later, even in cases managed non-operatively, owing to significant callus formation. Additionally, inadvertent clavicle shortening during fixation may compress the plexus and vessels, potentially leading to secondary thoracic outlet syndrome (TOS). This was evident in cases where the clavicle had been shortened by more than 0.5 cm [8]. Although iatrogenic neurovascular complications are rare, they can have severe consequences, including limb-threatening or fatal outcomes. This risk persists despite clavicle surgery being intended to address non-life-threatening issues [9,10].

The timing of initial fixation appears to influence the risk of neurovascular injury, with a peak incidence observed between two and three weeks post-injury. Delayed fixation of clavicle fractures increases the risk of neurovascular injury, attributed to scar tissue tethering the neurovascular structures to the fracture site. This occurrence likely results from traction injury during fracture mobilization, owing to nerve and vessel adherence to the fracture fragments. The extent of fibrotic reaction correlates with fracture severity, with more complex fractures associated with increased neurovascular structure tethering and compression [11]. The predominant plexus injury pattern involves traction or rupture of the C5/C6 nerve roots or upper trunk, particularly in cases of more comminuted fractures [12].

We present a case series to highlight the neurological and vascular complications associated with clavicle fractures and highlight the importance of careful assessment and decision-making in managing clavicle fractures.

## Case presentation

Neurovascular complications from clavicle fractures present significant challenges. Here, we present three cases illustrating distinct complications: venous obstruction, arterial thoracic outlet syndrome, and acute brachial plexus injury. These cases underscore the progressive nature of such complications and their significant impact on patient quality of life, highlighting the critical role of comprehensive assessments, advanced imaging, and timely surgical intervention to optimize outcomes. Early vigilance is essential for the effective management of potential neurovascular risks associated with clavicle fractures.

Case 1: Venous obstruction

**Figure 1:**
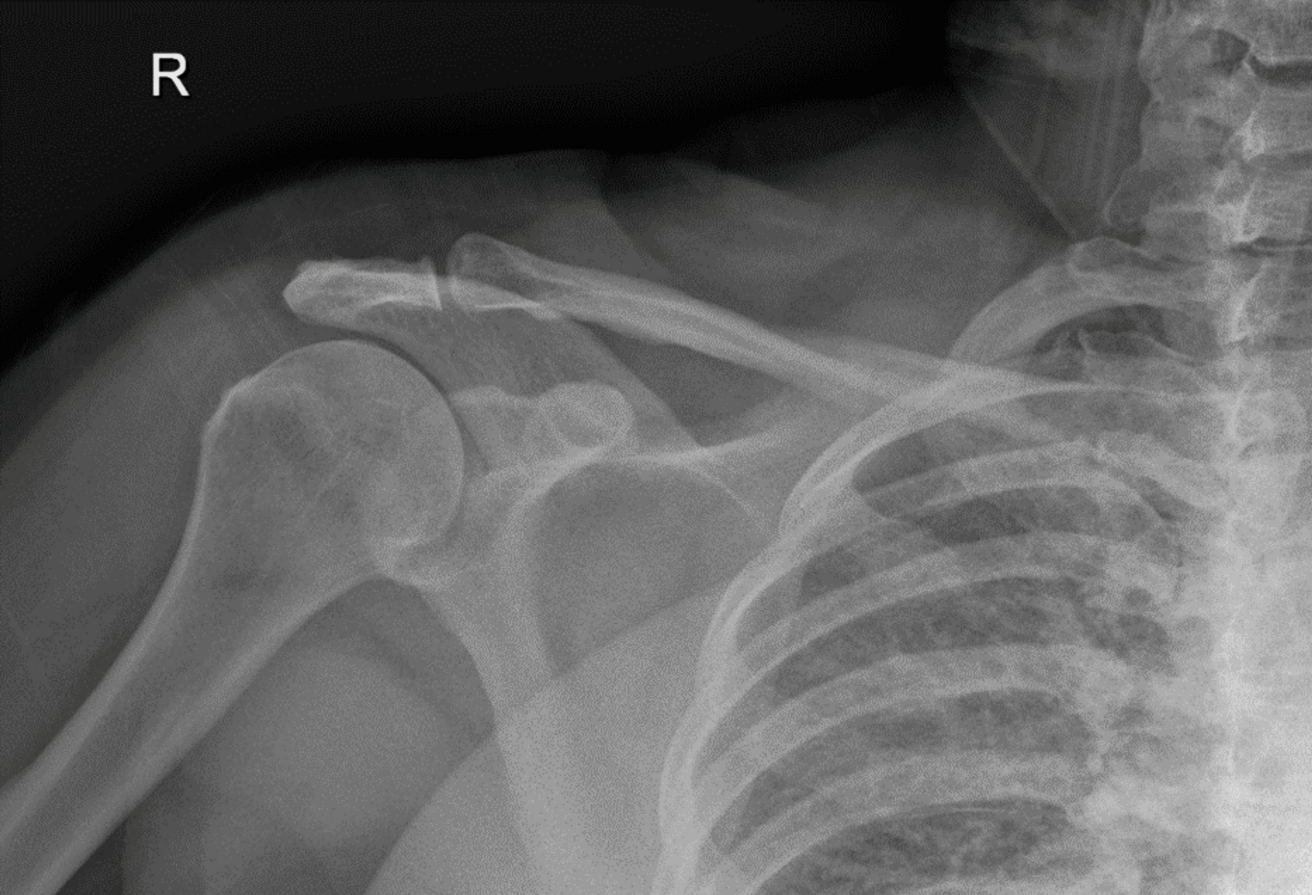
Case 1: Initial shoulder X-ray showing a right midshaft clavicle fracture, which appears to be non-displaced

The first patient is a 48-year-old woman who sustained a right shoulder injury after falling while riding her bike downhill. Upon presentation to the local emergency department (ED), she has a pins and needles sensation in her right arm, along with neck pain. Neurological assessment yielded normal results. A trauma series CT scan of the head, neck, and thorax revealed a scapular fracture, as well as fourth and seventh undisplaced ipsilateral rib fractures. Additionally, a midshaft clavicle fracture was identified on a plain X-ray (Figure 1). The patient had previously been diagnosed with multiple disc protrusions from C3 to C7, with subsequent MRI showing no worsening. Following evaluation, she was discharged from the ED with an arm sling and scheduled for follow-up in the orthopedic department.

**Figure 2:**
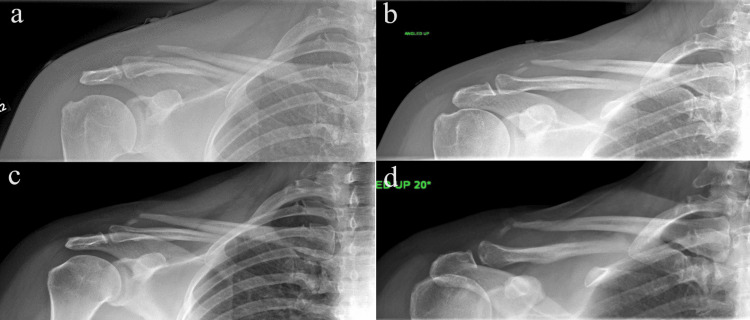
Case 1: Two-week (a and b) and three-month (c and d) follow-up X-ray revealing additional displacement and shortening, with medialization of the lateral clavicle fragment a and c depict the anterior-posterior shoulder X-ray, while b and d show the 20° cephalic tilt shoulder X-ray, highlighting notable medialization and shortening.

**Figure 3:**
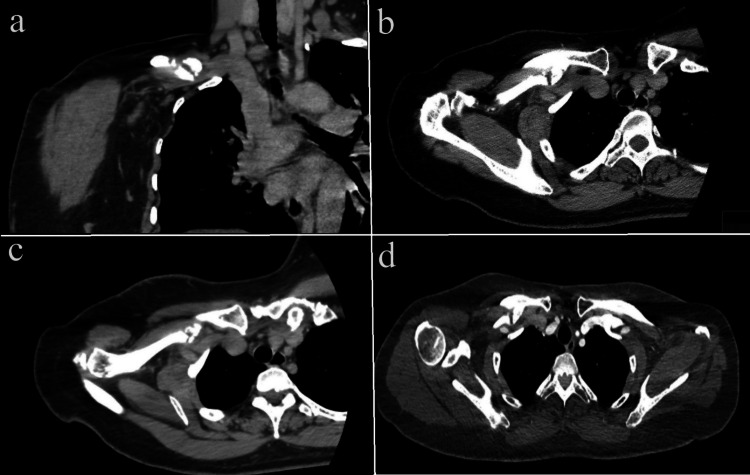
Case 1: Pre-operative shoulder CT scan depicting nonunion and abundant callus at the fracture site, with the axillary vein closely situated a (coronal cut) and b and c (axial cut) highlight these features. d displays the CT angiogram, indicating compression of the axillary vein by the lateral segment at the fracture site.

Upon review in the fracture clinic after two weeks, the fracture was found to have displaced, yet the decision was made to continue non-operative treatment. However, at the three-month follow-up, the radiograph revealed further displacement and shortening, with medialization of the lateral clavicle fragment (Figure 2). The patient, who had pre-existing right-side radiculopathy due to multiple disc prolapses from C3 to C7, reported worsening of symptoms post-injury. She experienced heaviness in her forearm, altered sensation, and tingling extending into her index and thumb, exacerbated by shoulder extension and abduction, hanging off the arm, and elbow flexion to 45°. These symptoms, described as electric shock sensations down the forearm and into the right thumb, index finger, and left thumb, intensified with neck lateral rotation and side flexion. Despite demonstrating good shoulder movement with minimal pain, a CT scan at three months showed nonunion with florid callus (Figure 3). Concerns about fracture compression on neurovascular structures prompted a pre-operative CT angiogram, revealing axillary vein compression. Consequently, the patient was referred to a tertiary center with vascular support.

**Figure 4:**
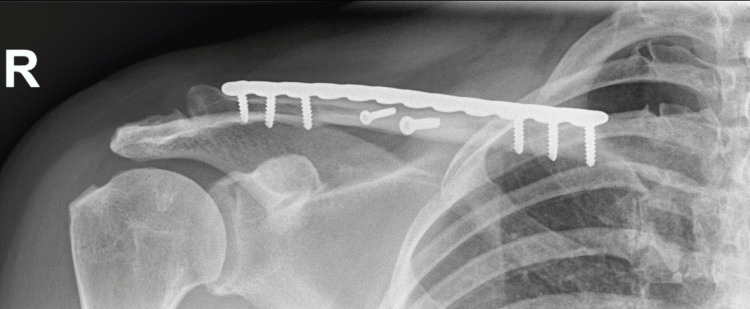
Case 1: Post-operative shoulder X-ray illustrating a healed fracture with a satisfactory position

The patient underwent clavicle fixation and plexus exploration as a combined orthopedic, plastic, vascular, and cardiothoracic case. The fracture was mobilized, and the neurovascular structures were explored and protected. The clavicle was fixed with a plate and screw construct (Figure 4). The fracture united by six weeks, the patient's vascular symptoms resolved, and her radiculopathy improved to pre-injury level.

Case 2: Arterial thoracic outlet syndrome

**Figure 5:**
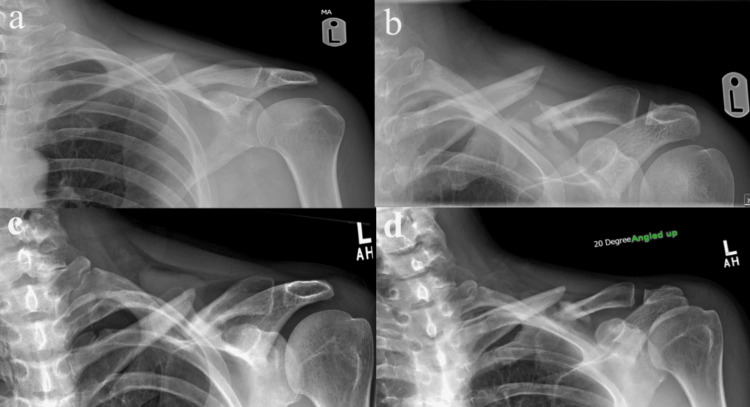
Case 2: Left shoulder X-ray, including initial AP (a) and cephalic tilt (b), revealing significant displacement and shortening, and six-week (three months) (c and d) follow-up X-ray indicating nonunion AP: anterior-posterior

The second case is a 51-year-old male patient who has a desk-based job but is very active and performs a lot of outdoor activities. He attended the ED after falling from a bike and had an initial X-ray performed that revealed a left midshaft clavicle fracture (Figure 5). There was no neurovascular deficit, so he was treated with a sling and discharged with follow-up in the fracture clinic. During his initial review, there was a discussion regarding operative versus non-operative management, but the extent of fracture displacement was underestimated. Consequently, a decision was made for non-operative management. However, at six weeks, the patient continued to struggle, prompting a referral to the shoulder service at three months.

**Figure 6:**
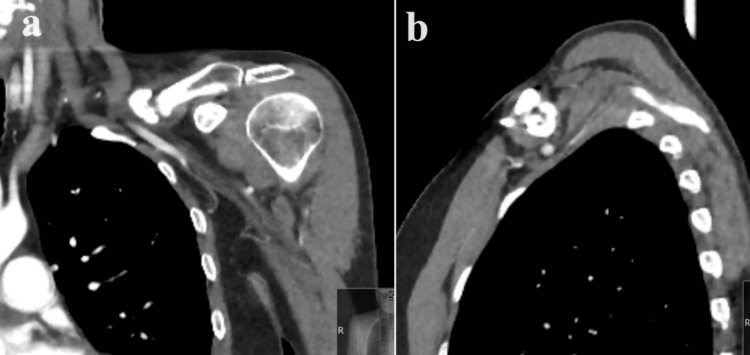
Case 2: CT angiogram illustrating florid callus formation, resulting in a marked reduction in the subclavicular space a shows the coronal view, while b shows the sagittal view.

Clinically, he experienced difficulty lifting his arm above head height and frequently developed numbness and tingling in all his fingers. He reported episodes where his hand would turn white and feel cold. There was noticeable scapular protrusion due to clavicular protrusion. Neurological assessment, pulses, and venous return were normal at rest. He did exhibit a degree of radial-radial delay, his radial pulse disappeared upon elevation of the arm, and he tested positive for Adson's test for arterial occlusion. A CT angiogram showed florid callus formation, significantly reducing the subclavicular space. The callus was located within 5 mm of the artery, clearly causing his vascular thoracic outlet syndrome (Figure 6).

**Figure 7:**
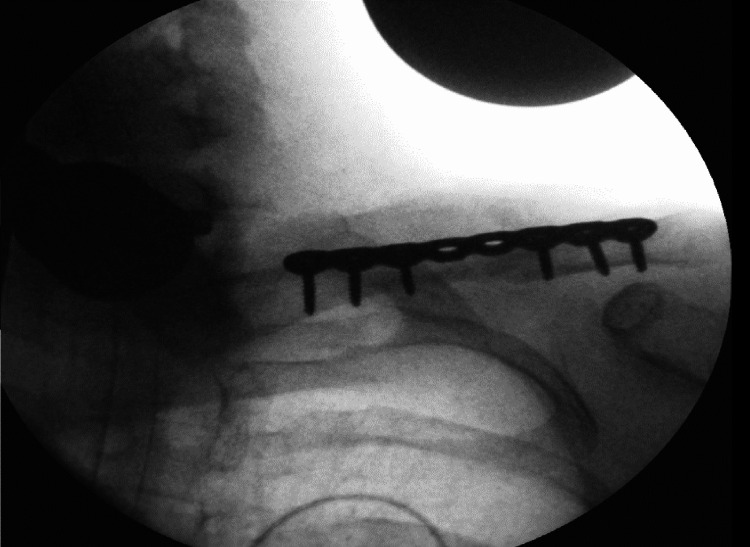
Case 2: Intra-operative fluoroscopic evaluation revealing good reduction of the left clavicle midshaft fracture

The patient underwent open reduction, grafting, and clavicle fixation performed by an orthopedic surgeon, with the presence of a vascular surgeon. Clavicle fixation was achieved using a standard plate and screw construct (Figure 7). Following surgery, the patient's thoracic outlet symptoms resolved.

Case 3: Acute brachial plexus injury

**Figure 8:**
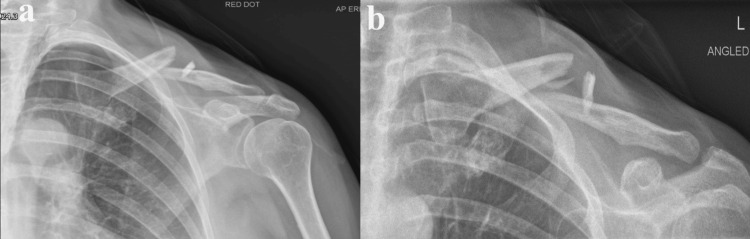
Case 3: Initial left shoulder X-ray exhibiting significant displacement, shortening, and a butterfly fragment a displays the AP view, while b shows the cephalic tilt view. AP: anterior-posterior

The third case is a 67-year-old female patient who fell down a few stairs, sustaining a shoulder injury. She sought assessment at a nearby hospital ED, where a plain X-ray revealed a left clavicle midshaft comminuted fracture (Figure 8). The patient exhibited paresthesia and no motor function in her left arm. Despite evidence of severe plexus injury, she was managed non-operatively and discharged with a sling, with a follow-up appointment scheduled for a few weeks later. Unfortunately, her symptoms did not improve, and swelling developed in her arm and fingers. Consequently, she presented to our ED and was admitted for further evaluation.

**Figure 9:**
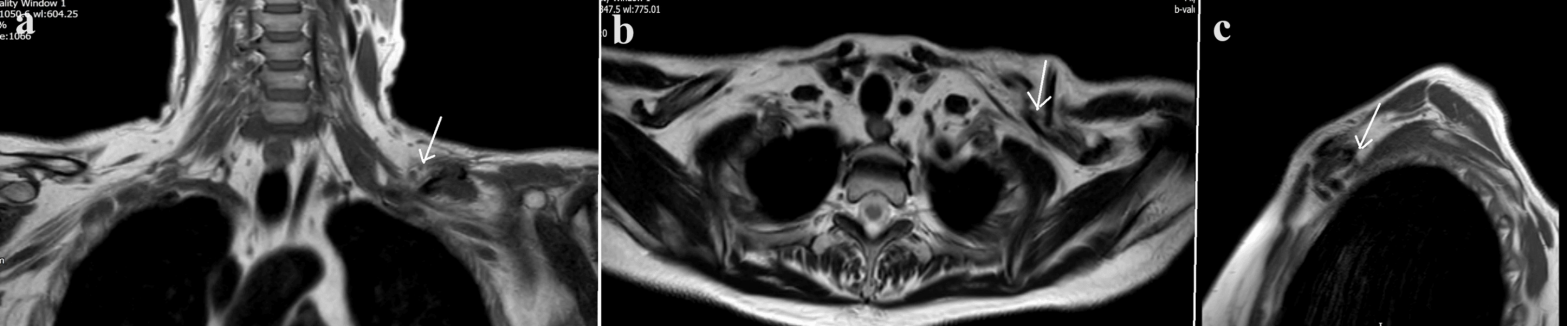
Case 3: Plexus MRI revealing direct compression of the plexus by both the lateral fracture end and the butterfly fragment a, b, and c show the coronal, axial, and coronal views, respectively. The arrow indicates the site of compression.

Clinically, she exhibited normal vascular pulses and venous return. However, there was a complete absence of motor and sensory function in the left arm and hand. Nerve function in the shoulder region was challenging to assess, but there was no function observed in the musculocutaneous, median, ulnar, and radial nerves. An MRI scan of the plexus revealed direct compression of the plexus by both the lateral fracture end and the butterfly fragment (Figure 9). Early involvement of the regional peripheral nerve injuries unit was initiated, and acute clavicle fixation was recommended.

**Figure 10:**
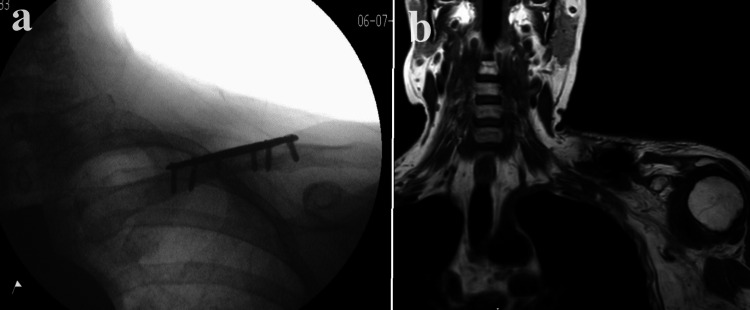
Case 3: a displaying intra-operative fluoroscopic evaluation, indicating satisfactory reduction, and b showing post-operative MRI, revealing the plexus appearing in continuity

The patient underwent fracture fixation using a plate and screw construct two weeks after the injury. The surgery proceeded without intra-operative complications, achieving satisfactory fixation. The peri-plexus fat remained undisturbed, and due to the acute nature of the injury, neurolysis was unnecessary. A post-operative MRI showed that the plexus remained in continuity (Figure 10). Management of the plexus injury involved expectant management with regular hand therapy, along with the use of splints and a sling.

The fracture had healed by two months. Nerve conduction studies (NCS) revealed moderately severe axonotmesis affecting the posterior, medial, and lateral cords. At the three-month follow-up, signs of recovery for the brachial plexus injury were evident, with restored deltoid and biceps function, along with mild improvement in forearm pronation and supination. Additionally, she regained some flicking movement in her fingers.

Although the patient exhibited progressive improvement during serial follow-up, she continued to experience limited shoulder movement and discomfort at 14 months postoperatively, along with tenderness around the shoulder. Upon examination, shoulder forward flexion was limited to 150°, abduction to 130°, external rotation to 50°, and internal rotation to the waist level. Dysaesthesia was noted along C6-C7 dermatomes, and weakness was observed in finger flexion (C8). Wrist movement was limited, with dorsiflexion of 10° and volar flexion of 20°, and stiffness was noted in the fingers at the proximal interphalangeal joint (PIPJ). Six months later, at the 20-month follow-up, further improvement was observed in elbow, wrist, and hand function, albeit with residual finger stiffness. Given the severity of the injury, her recovery has been remarkable.

## Discussion

The neurovascular assessment is imperative for evaluating any fracture at presentation, post-intervention, and during follow-up. Due to the clavicle's proximity to the brachial plexus and subclavian vessels, a thorough evaluation of both the neurological and vascular status of the entire upper limb is essential for surgeons managing clavicle fractures.

Venous injury may occasionally lead to significant bleeding, evident upon drill removal from the medial plate hole, indicating potential penetration of the thin-walled subclavian vein adhering to the posterior periosteum. Negative intrathoracic pressure can create a suction effect in the subclavian vein, drawing air through any breach in the vessel wall, potentially causing air embolism [13]. Deep vein thrombosis (DVT) commonly accompanies non-operative clavicle fracture management and often correlates with underlying conditions such as thoracic outlet syndrome, as seen in Paget-Schroetter syndrome (PSS). This syndrome manifests as effort-induced thrombosis of the axillary and subclavian veins due to thoracic outlet compression [14,15].

Reports of arterial injury associated with clavicle fractures often involve prominent screws in the medial two-thirds of the clavicle, leading to direct puncture or vessel abrasion during arm movement. These injuries typically manifest years after surgery as pseudoaneurysms, which may remain asymptomatic before presenting with limb weakness or embolic symptoms. Treatment usually entails vascular stent or graft procedures [16,17]. Arteriovenous fistulae, stemming from penetrating injuries, can lead to venous hypertension and limb-threatening ischemia if untreated, often manifesting as a pulsatile supraclavicular mass and ipsilateral arm weakness post-clavicle fixation. Early intervention is crucial to prevent severe complications [18].

The brachial plexus is susceptible to injury during trauma or clavicle surgery and prone to damage from traction, compression, and entrapment. It often becomes tethered to fracture fragments and callus during the healing process, resulting in traction force on the plexus during bone fragment reduction [19]. However, instances of brachial plexus palsy have been observed during or after both conservative and surgical treatments, occasionally appearing months to years following the fracture. While delayed-onset brachial plexus lesions post-clavicle fractures are rare, the gradual onset of palsy within a month after the fracture, especially in patients initially without neurovascular symptoms, is exceedingly uncommon [6].

Jeyaseelan et al. documented 21 cases of brachial plexus injury following delayed fixation of displaced clavicle fractures [20]. They observed that 24% of cases were fixed in a shortened position (>0.5 cm), contributing to the compressive mechanism on the plexus. Additionally, hypertrophic callus and malunited fractures can exacerbate compression [21]. While minimal sub-periosteal dissection is generally recommended for treating bone nonunion, Jeyaseelan et al. suggested thorough release of adherent soft tissues from the inferior clavicle before fragment mobilization in cases of delayed fixation. Della Santa et al. identified 16 cases of late brachial plexus lesions over 20 years, with only one attributed to direct compression of a displaced bone fragment [6].

The displacement of the middle-third clavicle fractures might represent specific characteristics associated with brachial plexus palsy following clavicle fractures, even in cases of simple fractures. Surgical findings indicated a ruptured subclavius muscle and the presence of granulation tissue at the distal clavicle fracture site. Granulation tissue could potentially contribute to subacute brachial plexus palsy. This underscores the importance of considering granulation tissue as a factor, even when no evident changes in fracture displacement or callus formation are visible on X-ray. Treatment options ranged from decompression of the brachial plexus combined with open reduction and internal fixation (ORIF) of the clavicle fracture to partial resection of the clavicle as an alternative approach [22]. Neurolysis often leads to rapid pain relief, particularly in cases where no prior improvement was observed, highlighting the possibility of neurostenalgia [23]. This syndrome results from a nerve lesion persisting in an anatomically intact nerve, often due to entrapment, distortion, or ischemia. Neurolysis can alleviate pain and restore neurological function by removing the causal factor, such as scar tissue. The suprascapular nerve's direct involvement appears to be a common occurrence, likely due to its superficial location behind the clavicle, making it more susceptible to scar tissue effects [24,25].

Comprehensive soft tissue dissection and avoidance of clavicle shortening during fixation are recommended to reduce the risk of neurovascular injury. Midshaft clavicle fractures fixed between two and four weeks post-injury pose a small yet significant risk of brachial plexus injury, especially in cases of increased fracture comminution or clavicle shortening. Initial shortening of the clavicle by 20 mm or more is considered a risk factor for nonunion.

Thoracic outlet syndrome (TOS) occurs due to the compression of either nerves (brachial plexus) or blood vessels (subclavian/axillary vein or, less commonly, subclavian/axillary artery) at the thoracic outlet [26]. While the scalene triangle is typically the primary site of compression in all TOS forms, the costoclavicular space is predominantly affected in clavicle fracture cases. Abundant callus and displaced fractures can further narrow the costoclavicular space, exacerbating TOS [27]. However, TOS symptoms, often positional, may overlap with persistent shoulder and upper limb symptoms in patients with clavicle fractures, potentially leading to missed or delayed diagnoses due to the absence of findings on routine neurovascular examinations. Hence, maintaining a high level of suspicion and conducting specialized clinical tests and imaging are essential for an accurate diagnosis.

In our series, all fractures occurred at the midshaft, and initially, non-operative treatment was considered. In the first case, despite initially opting for non-operative management due to acceptable displacement of the clavicle fracture, reassessment at three months revealed further displacement and shortening, prompting a change in the treatment plan. The patient's pre-existing radiculopathy from multiple disc prolapses complicates the recognition of neurological symptoms. However, the exacerbation of her pre-existing symptoms alongside the onset of new symptoms raises suspicion for nerve involvement, necessitating a comprehensive neurological assessment and appropriate diagnostic testing. In our case, a CT angiogram demonstrated axillary vein compression, guiding pre-operative planning and management. While MRI could provide additional details about brachial plexus compression, it was not conducted in our case. Multidisciplinary management resulted in fracture union and resolution of vascular symptoms, underscoring the importance of thorough evaluation and prompt recognition of neurovascular complications in such cases.

In the second case, the patient initially underwent non-operative treatment despite significant fracture displacement, which was initially underestimated. However, at the three-month mark, signs of nonunion became apparent, accompanied by persistent functional impairment and numbness in the fingers, along with episodes of hand pallor and coldness. Despite normal initial neurology, pulses, and venous return at rest, the presence of associated thoracic outlet syndrome warranted a high index of suspicion. However, delayed radial-radial pulse and a positive Adson's test for arterial occlusion prompted further investigation with a CT angiogram, which confirmed a reduction in the subclavicular space due to a florid callus located within 5 mm of the subclavian artery, conclusively identifying the cause of the patient's vascular thoracic outlet syndrome.

The third case presented a scenario of a comminuted midshaft clavicle fracture accompanied by a brachial plexus injury. Operative intervention was pursued to address the clavicle fracture and decompress the brachial plexus from the displaced fracture fragments, while the brachial plexus injury itself was managed conservatively through observation for signs of recovery. Although the patient demonstrated remarkable improvement over time, residual deficits persisted, highlighting the enduring impact of such injuries. At 14 months post-operatively, the patient continued to experience limitations in shoulder movement and discomfort, with examination findings revealing ongoing dysaesthesia, weakness, and stiffness in specific dermatomes and joints. This case underscores the potential severity of brachial plexus injuries associated with clavicle fractures and emphasizes the importance of early recognition and intervention.

In cases of comminuted fractures, particularly those showing significant initial displacement and further displacement during follow-up, along with notable shortening and presentation within two to four weeks post-injury, a thorough investigation of potential neurovascular complications is imperative [28]. Utilizing diagnostic tools such as CT angiogram, MRI, and nerve conduction studies (NCS) becomes essential to accurately assess the extent of neurovascular involvement and in pre-operative planning. These comprehensive assessments enable healthcare providers to promptly identify and address any underlying neurovascular issues, ultimately optimizing patient care and minimizing the risk of long-term complications associated with such fractures.

The presented cases offer valuable learning points: brachial plexus compression might occur immediately or develop over time with conservative treatment of displaced clavicle fractures, presenting as various neurological symptoms. This underscores the importance of closely monitoring patients for any signs of neurological compromise during the healing process. Therefore, patients with such fracture types should undergo routine monitoring to detect any development of brachial plexopathy. In cases where patients present with neurological symptoms, prompt evaluation using appropriate diagnostic tools such as positional MRI and electromyography/nerve conduction velocity (EMG/NCV) testing is essential for timely diagnosis and intervention.

These insights underscore the need for meticulous clinical assessment and proactive management strategies to mitigate potential neurovascular complications associated with displaced midshaft clavicle fractures. By incorporating these principles into clinical practice, healthcare providers can optimize patient outcomes and reduce the risk of long-term sequelae.

As a summary, the following factors are considered high risk for iatrogenic neurovascular injury with clavicle fixation: a medial fracture, comminution, a two- to four-week delay from the time of injury, or nonunion, malunion, and revision cases. Therefore, for these scenarios, a recommendation is provided in Table 1.

**Table 1:** Recommendations to minimize the risk of iatrogenic neurovascular complications associated with clavicle fracture fixation NCS: nerve conduction studies, TOS: thoracic outlet syndrome

	Recommendations
Pre-operative	1. Obtain a pre-operative 3D CT angiogram to identify the exact location of neurovascular structures relative to the fracture and identify excessive callus.
	2. Communicate pre-operatively with anesthesiologists and vascular, cardiothoracic, and plastic surgeons for potential needs during surgery or considering referral of high-risk patients with available such services.
	3. Consider pre-operative NCS or MRI to identify plexus injury.
	4. Consider delaying surgery if the presentation is between two and four weeks.
Intra-operative	1. Perform sufficient sub-periosteal dissection from the medial and inferior clavicle to release any adherent soft tissues from the fracture site.
	2. Avoid aggressive traction during reduction to prevent traction injury.
	3. Avoid clavicle shortening as this makes the neurovascular tissue more prone to compression and increases the incidence of TOS.
	4. Exercise caution when applying retractors under the clavicle.
	5. Use a sharp drill to avoid applying pressure on the drill during reaming of the far cortex, which may lead to penetration of underlying structures.
	6. Consider the design of the clavicle plate and the direction of screws on the plate, with the safest screw position on the third of the clavicle being from superior to inferior and in the middle third being from anterior to posterior.
	7. Caution with screw length, especially when screws are applied eccentrically, as fluoroscopic evaluation can be misleading.

## Conclusions

Neurovascular complications associated with clavicular shaft fractures can manifest at presentation, develop gradually over time, or potentially be iatrogenically induced. Conducting a thorough neurovascular examination and, when warranted, pursuing further investigation through modalities such as CT angiogram, MRI, and nerve conduction studies (NCS) are crucial for early diagnosis and pre-operative planning. This comprehensive approach enhances patient outcomes by facilitating timely intervention and addressing any underlying neurovascular issues associated with the fracture.

High-risk factors for neurovascular injury in clavicle fixation include medial fracture, comminution, two- to four-week injury delay, nonunion, malunion, and revisions. Preoperative measures include performing proper imaging evaluation, multidisciplinary communication, and possible surgery delay. Intraoperative precautions involve meticulous dissection, traction avoidance, and screw placement awareness.
